# Clinical *Candida albicans* Vaginal Isolates and a Laboratory Strain Show Divergent Behaviors during Macrophage Interactions

**DOI:** 10.1128/mSphere.00393-20

**Published:** 2020-08-19

**Authors:** Franziska Gerwien, Christine Dunker, Philipp Brandt, Enrico Garbe, Ilse D. Jacobsen, Slavena Vylkova

**Affiliations:** a Septomics Research Center, Friedrich Schiller University and Leibniz Institute for Natural Product Research and Infection Biology–Hans Knoell Institute, Jena, Germany; b Research Group Microbial Immunology, Leibniz Institute for Natural Product Research and Infection Biology–Hans Knoell Institute, Jena, Germany; University of Georgia

**Keywords:** *Candida albicans*, vulvovaginal candidiasis, macrophages, cell wall

## Abstract

Typically, established lab strains are widely used to study host-pathogen interactions. However, to better reflect the infection process, the experimental use of clinical isolates has come more into focus. Here, we analyzed the interaction of multiple vaginal isolates of the opportunistic fungal pathogen Candida albicans, the most common cause of vulvovaginal candidiasis in women, with key players of the host immune system: macrophages. We tested several strains isolated from asymptomatic or symptomatic women with acute and recurrent infections. While all clinical strains showed a response similar to the commonly used lab strain SC5314 in various *in vitro* assays, they displayed remarkable differences during interaction with macrophages. This coincided with significantly reduced β-glucan exposure on the cell surface, which appeared to be a shared property among the tested vaginal strains for yeast extract/peptone/dextrose-grown cells, which is partly lost when the isolates faced vaginal niche-like nutrient conditions. However, macrophage damage, survival of phagocytosis, and filamentation capacities were highly strain-specific. These results highlight the high heterogeneity of C. albicans strains in host-pathogen interactions, which have to be taken into account to bridge the gap between laboratory-gained data and disease-related outcomes in an actual patient.

**IMPORTANCE** Vulvovaginal candidiasis is one of the most common fungal infections in humans with Candida albicans as the major causative agent. This study is the first to compare clinical vaginal isolates of defined patient groups in their interaction with macrophages, highlighting the vastly different outcomes in comparison to a laboratory strain using commonly applied virulence-determining assays.

## Abstract (precis)

Vulvovaginal candidiasis is one of the most common fungal infections in humans with Candida albicans as the major causative agent. This study is the first to compare clinical vaginal isolates of defined patient groups in their interaction with macrophages, highlighting the vastly different outcomes in comparison to a laboratory strain using commonly applied virulence-determining assays.

## OBSERVATION

Candida albicans is an opportunistic fungal pathogen and a normal colonizer of mucosa of the gut, the oral cavity, and the vulvovaginal tract. When the balance of the microbial flora is disrupted or the immune defenses are compromised, it can become pathogenic, often causing recurrent disease in susceptible individuals (1). Symptomatic infections in the female reproductive tract, termed vulvovaginal candidiasis (VVC), typically occur in otherwise healthy women. Fungal overgrowth, subsequent epithelial invasion, and immune cell infiltration lead to inflammatory symptoms like vaginal itching, burning, and pain (2). Albeit nonlethal, this disease affects 75% of all women at least once in their lifetime (3), while recurrent VVC (RVVC; defined as >3 episodes per year) affects about 8% of all women (4). These clinical representations diminish life quality and cause high costs in the global health system (5).

VVC is a multifactorial hyperinflammatory disorder with several known risk factors from the host side (antibiotic treatment, imbalance in vaginal microbiome, sexual activity, high estrogen levels, pregnancy, and low lactate levels), whereas the reasons for RVVC remain largely unknown (6). In the course of infection, C. albicans exploits one of its key virulence attributes: the ability to form hyphae. The filamenting fungus breaches epithelial barriers, and as a first line of defense phagocytic immune cells are recruited in vast numbers to mediate clearance. Elevated Th17-mediated cytokine secretion (interleukin-22 [IL-22], Il-17A, and IL-17F) and inflammasome activation, followed by IL-1β cleavage, also accompanies this process of hyperinflammatory immune cell infiltration into the vaginal tissues, which is largely responsible for the observed clinical symptoms (7). In this context, nutritional prerequisites have been shown to play an important role in modeling the fungal cell wall architecture and subsequent immune cell recognition (8, 9). In particular, lactate, a predominant carbon source in the vaginal tract (10) has been shown to influence host pathogen interaction and infection outcomes (11–13). Hence, we were particularly interested in studying host-pathogen interactions with strains that have not been extensively propagated in laboratories and come directly from a host niche, using a macrophage cell line as a feasible tool. For this purpose, we compared the commonly used laboratory C. albicans strain SC5314 to multiple clinical vaginal isolates from three defined patient groups: (i) asymptomatic C. albicans colonization, (ii) VVC, or (iii) RVVC. We observed that during macrophage encounter the isolates behave group-independently different than SC5314, which might be associated with their various capabilities to filament. However, fungal cell wall architecture, while vastly uniform for all tested vaginal isolates, is remarkably different from SC5314 for YPD (yeast extract, peptone, dextrose)-grown cells. Growth in the lactate containing vaginal simulating media unveiled partly group-specific differences in cell wall composition between the isolates, specifically in chitin and β-glucan exposure. Thus, the differences in cell surface architecture might lead to altered initiation of an immune response in the corresponding host niche.

### Vaginal isolates show great variability in macrophage interaction.

**FIG 1:**
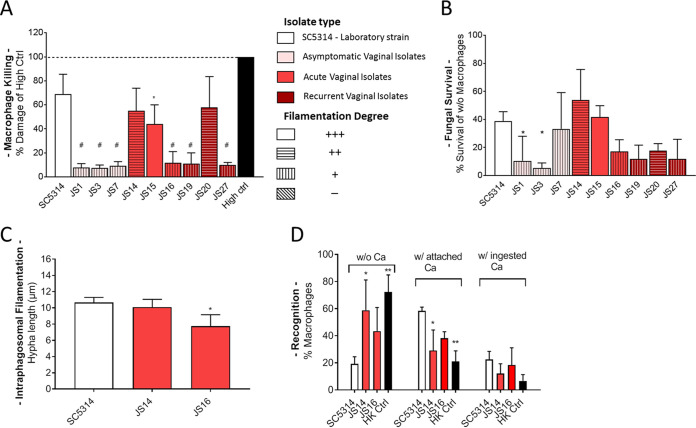
J774.1 macrophage interaction with C. albicans vaginal isolates. Color coding indicates Isolate type. (A) Macrophage killing (measured via LDH release) following infection with the selected strains. Damage is displayed as the percentage of Triton-X-treated high control (100% macrophage lysis) at 24 h postinfection. Uninfected macrophage control results were subtracted beforehand. Five biological replicates in technical triplicates were conducted. The degree of hyphal formation (DMEM with macrophages, 24 h, 37°C) is indicated by the pattern of the bar: +++, SC5314-like hypha length with branching; ++, moderate hyphae length with marginal branching; +, short hyphae; −, no hyphae or sporadic germ tubes. (B) Fungal killing was assessed for SC5314 and all tested isolates after 24 h by calculating the ratio of *Candida* microcolonies using the following formula: (colonies in the presence of macrophages/colonies in DMEM) × 100. Three biological replicates were conducted. The degree of hyphal formation (DMEM with macrophages, 24 h, 37°C) is indicated by pattern of the bar: +++, SC5314-like hypha length with branching; ++, moderate hyphae length with marginal branching; +, short hyphae; –, no hyphae or sporadic germ tubes. (C) Intraphagosomal filamentation was assessed for SC5314, JS14, and JS16 by measuring the hyphal lengths of 100 phagocytosed yeasts at 1 h postinfection by fluorescence microscopy and differential staining (phagocytosed *Candida* cells: ConA negative and CFW positive). The presented data are from three biological replicates. (D) Recognition by macrophages was assessed 30 min postinfection for SC5314, JS14, or JS16 strains and presented as the percentage of macrophages nonassociated with fungal cells (w/o Ca), with attached fungal cells (w/attached Ca), or with ingested fungal cells (w/ingested Ca). Heat-killed *Candida* cells (HK Ctrl) were included as control with known diminished recognition due to induced cell wall aberrations. Three biological replicates were conducted. For statistical analysis, a one-way analysis of variance (ANOVA) was performed, followed by Dunnett’s multiple-comparison test (*, *P* < 0.05; **, *P* < 0.01; #, *P* < 0.0001 [compared to SC5314]).

Macrophages, similarly to polymorphonuclear leukocytes, recognize, phagocytose, and subsequently kill invading pathogens as part of their role in the innate immune response. C. albicans has been shown to be able to escape phagocytosis via hyphae formation (14). Therefore, we tested the ability of the clinical isolates to filament in various hyphae-inducing *in vitro* conditions (see Fig. S1 in the supplemental material) and observed great variability between the isolates with no clear pattern within a specific patient group. Likewise, various degrees of filamentation were noted when the vaginal strains were cocultured with macrophages in Dulbecco's modified Eagle’s medium (DMEM), with the majority of the isolates showing prominent defects in hyphal growth compared to the SC5314 control (Fig. 1A; see also Fig. S2). Overall, a clear association between filamentation and macrophage damage was observed: strains with robust hyphal growth were more likely to cause immune cell damage similar to the SC5314 strain, whereas less filamenting strains failed to induce a SC5314-like LDH release (Fig. 1A). Consequently, the capacity to form filaments appeared to be tightly connected with fungal survival following confrontation with macrophages, with the exception of isolates JS7 and JS20 (Fig. 1B). Of particular interest were strains JS14 and JS16, both isolated from VVC patients, due to their opposing characteristics: while JS14 was able to filament, damage macrophages, and survive phagocytosis, JS16 was impaired in all of these aspects. Importantly, these two strains appeared to be nearly indistinguishable in the *in vitro* filamentation assays. Since both fungal recognition by the immune cells and the ability to filament within the phagosome can influence the outcome of infection, we chose these particular strains to test in detail their interaction with J774.1 cells. JS16 showed a slightly diminished intracellular hyphal length compared to the SC5314 laboratory strain (Fig. 1C), whereas JS14 was not as well recognized by macrophages (Fig. 1D). These mild phenotypes were rather surprising since they could not explain the gross differences in infection outcomes. In summary, the vaginal isolates react to macrophages in a patient group-independent manner, showing various filamentation defects that affect macrophage interaction. Infection outcome is highly strain specific and does not reflect pathogenicity-related grouping in a clinical setting.

10.1128/mSphere.00393-20.1 FIG S1 Filamentation of vaginal isolates in different hypha-inducing media. Color coding indicates the isolate type. Degree of hyphal formation (DMEM with macrophages, 24 h, 37°C) is indicated by pattern of the bar: ++++, hyphae length exceeds SC5314; +++, SC5314-like hypha length with branching; ++, moderate hyphae length with marginal branching; +, short hyphae; –, no hyphae or sporadic germ tubes. Incubation time was 6 h (liquid) or 2 days (solid). Composition of media: GlcNac (2% *N*-acetyl-d-glucosamine, 0.5% ammonium sulfate, 0.17% YNB), human serum (10% heat-inactivated human serum, 0.5% ammonium sulfate, 0.17% YNB, 2% glucose, 2% agar), or Spider (1% nutrient broth, 1% mannitol, 0.2% K_2_HPO_4_, 2% agar [pH 7.2]). Download FIG S1, TIF file, 0.7 MB. Copyright © 2020 Gerwien et al. 2020 Gerwien et al. This content is distributed under the terms of the Creative Commons Attribution 4.0 International license.

10.1128/mSphere.00393-20.2 FIG S2 Filamentation of vaginal isolates with J774.1 macrophages in DMEM. C. albicans strains were cocultured with J774.1 macrophages in DMEM (MOI of 5, 24 h). Pictures were taken after conducting the macrophage killing assay. Between the three infection-related groups (asymptomatic, acute, and recurrent isolates), various isolates showed group-independent filamentation defects. Scale bar, 100 μm. Download FIG S2, TIF file, 0.2 MB. Copyright © 2020 Gerwien et al. 2020 Gerwien et al. This content is distributed under the terms of the Creative Commons Attribution 4.0 International license.

### Vaginal isolates have different cell wall architecture in rich medium compared to SC5314.

**FIG 2:**
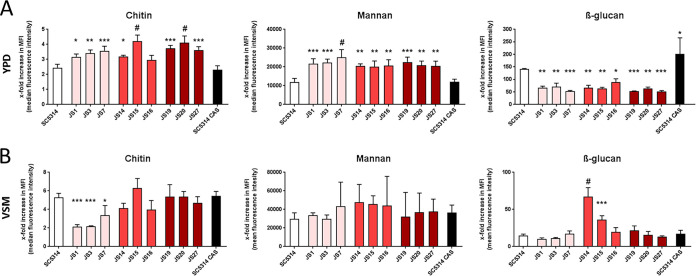
Cell wall architecture of SC5314 and C. albicans vaginal isolates. The levels of chitin, mannan, and β-glucan were measured as the *x*-fold increase of median fluorescence intensity (MFI) over an unstained control via differential staining and flow cytometry. Values are shown as means ± the standard deviations (*n* = 3). For statistical analysis, a one-way ANOVA was performed, followed by Dunnett’s multiple-comparison test (*, *P* < 0.05; **, *P* < 0.01; ***, *P* < 0.001; #, *P* < 0.0001 [compared to SC5314]). (A) Fungal cells were grown as an ON culture in YPD. A sublethal concentration of caspofungin (CAS; 0.625 ng/ml = 1/4 MIC_50_) was added to SC5314 as a positive control for β-glucan exposure. (B) Fungal cells were grown for 2 days in VSM.

The fungal cell wall composition plays an essential role in initial recognition by the immune cells with chitin, mannan, and β-glucan being the main components (15). It is known that fungal β-glucan is highly immuno-reactive (16) and β-glucan masking by mannan can inhibit fungal recognition and killing by macrophages (17, 18). Nutritional factors, such as lactate, can induce β-glucan masking mediated by the exoglucanase Xog1 presumably as a strategy to reduce the visibility of the commensal fungus to the immune system (19). Here, we compared surface exposure of the cell wall components of the vaginal isolates when grown either in commonly used laboratory rich medium (YPD) or in niche-specific vaginal simulating medium (VSM). Surprisingly, all vaginal isolates displayed significantly less β-glucan exposure (25 to 50% of the laboratory strain SC5314) and elevated mannan and chitin exposure when grown in YPD (Fig. 2A). However, when grown in VSM the total β-glucan MFI values were decreased for SC5314, compared to YPD, with similar intensities in strains of the asymptomatic and recurrent group (Fig. 2B). In VSM, there also appeared to be group-specific effects for asymptomatic strains (less chitin) and acute strains (more β-glucan) compared to SC5314 and the other tested strains. Since β-glucan masking in lactate containing media such as VSM has been reported to reduce immune visibility (19), our results imply specifically that the acute strains might be more immunoreactive. This would fit to the common view that clinical symptoms during acute vaginal infections are mostly accounted for host-driven hyperinflammatory response toward *Candida* colonization (7). The vaginal isolates might have lost their typical response pattern to the rich medium YPD (in contrast to SC5314), keeping their lactate-primed β-glucan masking constitutively active. However, upon exposure to environmental conditions representing the host niche, we noted group-specific patterns in β-glucan and chitin exposure, which might explain the differences in pathogenicity.

### Conclusion.

Altogether, these results suggest that vaginal clinical isolates are highly specialized to their host niche and likely even to the immunological and nutritional status of the individual they have been isolated from. In this form they might lose adaption capacity to environmental changes, as was seen with the filamentation defects during lab cultivation conditions and the altered cell wall arrangements, when exposed to the laboratory rich medium YPD. Tight adaption to the originating host niche might render clinical strains less flexible to the conditions used in typical laboratory tests but still highly specialized to persist in their host niche. Caution has to be applied since these strains might appear defective in commonly applied laboratory tests, and yet they are likely highly competent in the niche where they originate, as shown by their ability to persist and even cause disease. These observations highlight the importance of studying more niche-specific nutritional, immunological, and microbiome conditions.

### Macrophage infection assays.

For all macrophage experiments J774A.1 cells were cultivated in DMEM plus 10% fetal bovine serum at 37°C and 5% CO_2_. Macrophages were seeded in a 96-well plate (4 × 10^4^ cells/well for the macrophage killing assay or 1 × 10^4^ cells/well for the fungal survival assay) or in a 24-well plate (1 × 10^5^ cells/well, 24-well plate for the recognition and filamentation assay) and incubated overnight (ON). The medium was replaced with fresh DMEM, and the cells were infected with YPD-grown (30°C, 180 rpm) and washed *Candida* strains at a defined multiplicity of infection (MOI).

**(i) Macrophage killing assay.** For the macrophage killing assay, the macrophages were infected with 8 × 10^4^ *Candida* cells/well (MOI of 2), followed by incubation for 24 h. Then, 10 μl of 5% Triton X-100 was added (10 min, 37°C) to the noninfected high control to obtain full lysis. The plate was then centrifuged, and the supernatant was diluted 1:10 in phosphate-buffered saline (PBS). For lactate dehydrogenase (LDH) measurement, a cytotoxicity detection kit (Roche) was used according to the manufacturer’s protocol in technical triplicates. Emission at 542 nm was measured with a TECAN ElisaReader M200 (Software iControl). Noninfected macrophages were used as a negative control, and optical density values were subtracted from sample values.

**(ii) Fungal survival assay.** The fungal survival assay was performed in DMEM as described previously (20), with slight variations. A total of 2.5 × 10^4^ fungal cells/well (MOI of 2.5) were added to wells with or without macrophages, followed by five serial 1:5 dilutions. After 24 h of incubation, the cells were fixed, and microcolonies were counted using an inverse microscope in wells of the same dilution where a clear discrimination of the microcolonies was possible. Fungal survival was calculated as follows: (number of colonies in the presence of macrophages/number of colonies without macrophages) × 100.

**(iii) Recognition and filamentation assay.** For the recognition and filamentation assay, macrophages were infected with 5 × 10^5^ *Candida* cells (MOI of 5) and heat-killed (80°C, 20 min) SC5314 cells were added as the control, since heat treatment leads to poor recognition by the host cells due to disturbances in the cell wall architecture. The assay plate was incubated on ice for 30 min for synchronization of phagocytosis. Nonadhered C. albicans cells were washed away with DMEM. After incubation in DMEM for 30 min or 1 h, washing, and fixation (4% Histofix, 15 min, 37°C), nonphagocytosed fungal cells were stained with ConA-647 (concanavalin A conjugated to Alexa Fluor 647; 50 μg/ml in PBS, 30 min). After washing, cells were permeabilized with 0.5% Triton (5 min), and then counterstained with Calcofluor White (CFW; 35 μg/ml in 0.1 M Tris-HCl [pH 9], 20 min). Cells were washed with ddH_2_O (three times, 10 min), and samples were analyzed with a Zeiss Axio observer fluorescence microscope. The hyphal lengths of 100 phagocytosed cells (ConA negative, CFW positive) were measured. Recognition was determined by assessing the phagocytosis state of 200 macrophages via differential staining for having no association, attached (ConA positive, CFW positive), or ingested *Candida* cells (ConA negative, CFW positive).

### Staining of cell wall components.

Fungal cells were cultivated in either YPD (1 liter: 20 g peptone, 10 g yeast extract, 20 g glucose) or vaginal simulating media (1 liter: 2 g glucose, 0.16 g glycerol, 2 g lactic acid, 1 g acetic acid, 0.018 g bovine serum albumin [BSA], 0.4 g urea, 1.4 g KOH, 0.222 g CaCl_2_, 3.51 g NaCl [pH 4.2]), adapted from Vylkova and Lorenz (20). As previously described, a sublethal concentration of caspofungin (0.625 ng/ml = 1/4 MIC_50_) was added to SC5314 to obtains elevated β-glucan levels (21). Next, 1 × 10^6^ *Candida* cells were harvested, washed once in PBS, and fixed in 2% Histofix for 20 min and 600 rpm at room temperature. After an additional PBS washing step, the pellet was dissolved in 2% BSA/PBS and incubated for at least 10 min at 37°C to block unspecific binding. Simultaneous staining was performed for 1 h at 37°C and 100 rpm by the addition of 0.5 μl of primary anti-β-1,3-glucan antibody (Biosupplies, 1 mg/ml, stains β-1,3-glucan), 0.3 μl of ConA647 (Sigma, 5 mg/ml, stains mannan), and 7 μl of WGA-FITC (Sigma, 2 mg/ml, stains chitin) in 100 μl of 2% BSA/PBS per sample. After two washing steps with 2% BSA/PBS, 2 μl of secondary goat anti-mouse PE-Cy7 antibody (BioLegend, 0.2 mg/ml) was added in 100 μl of 2% BSA/PBS per sample for 30 min at 37°C and 100 rpm. After washing, the cells were resuspended in 2% BSA/PBS and analyzed with a FACSVerse (BD Biosciences) counting 10,000 events. Data analysis was performed using FlowJo 10.6.2 software.

10.1128/mSphere.00393-20.3 FIG S3 Gating strategy for staining of C. albicans cell wall components. C. albicans was incubated as indicated in YPD or VSM; stained for the respective cell wall components chitin, mannan, and β-glucan; and analyzed with a FACSVerse (BD Biosciences) counting 10,000 single yeast cells. Gating was performed to exclude debris and doublets. The median fluorescence intensity (MFI) was quantified and compared to an unstained control. Data analysis was performed using FlowJo 10.6.2 software. Exemplary measurements for YPD-grown strains SC5314 and JS14 are shown. Download FIG S3, TIF file, 0.4 MB. Copyright © 2020 Gerwien et al. 2020 Gerwien et al. This content is distributed under the terms of the Creative Commons Attribution 4.0 International license.
