# Supplementary figures and images for: Clinical Candida albicans Vaginal Isolates and a Laboratory Strain Show Divergent Behaviors during Macrophage Interactions
Source: mSphere. 2020 Aug 19;5(4):e00393-20. doi: 10.1128/mSphere.00393-20 (PMC7407065; doi:10.1128/mSphere.00393-20)

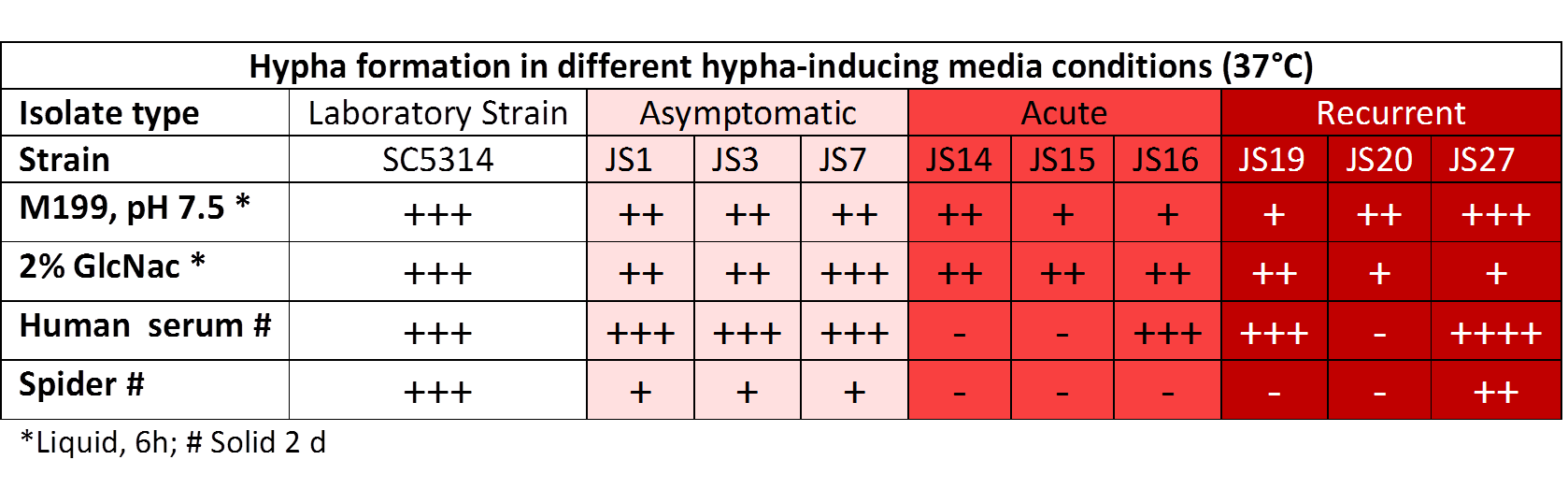

Supplement: FIG S1 [file mSphere.00393-20-sf001.tif]

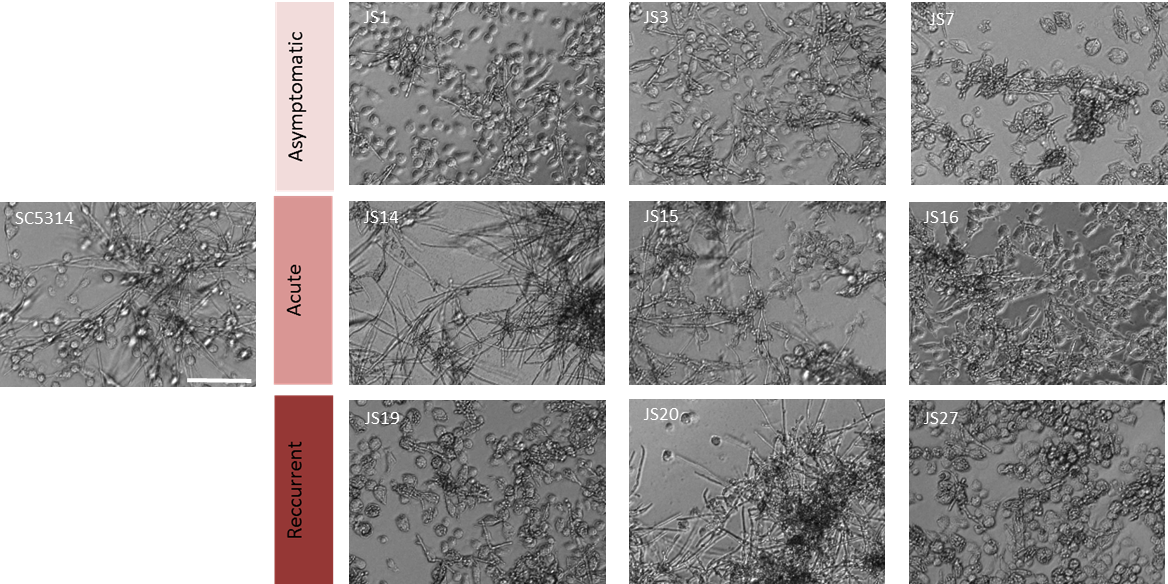

Supplement: FIG S2 [file mSphere.00393-20-sf002.tif]

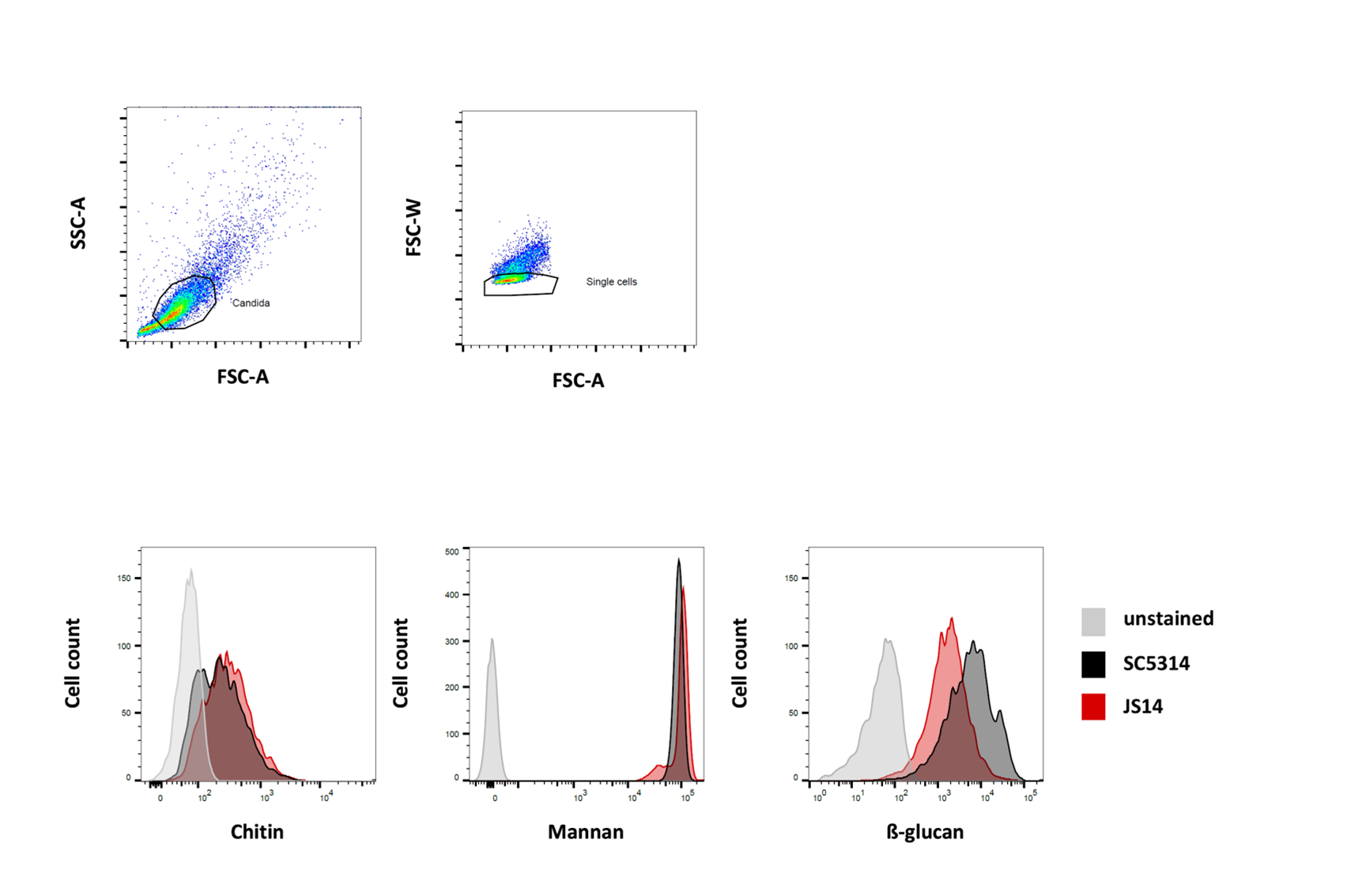

Supplement: FIG S3 [file mSphere.00393-20-sf003.tif]
